# Hormone-Dependent Cancers: New Aspects on Biochemistry and Molecular Pathology

**DOI:** 10.3390/ijms241310830

**Published:** 2023-06-29

**Authors:** Yasuhiro Miki

**Affiliations:** Department of Anatomic Pathology, Tohoku University Graduate School of Medicine, Sendai 980-8575, Miyagi, Japan; miki@patholo2.med.tohoku.ac.jp; Tel.: +81-22-717-8050; Fax: +81-22-717-8051

Hormones, especially steroids, are closely involved in the physiological functions and proliferation of various target tissues and have long been known to play a key role in the tumorigenesis or carcinogenesis of these target tissues. It is well known that sex steroid hormones, such as estrogen and androgen, have a close influence on the development and malignant behavior of endometrial, ovarian, and breast cancers in women and prostate cancer in men [1,2]. These cancers are hormone-dependent, and some of the mechanisms of hormone action have been identified as therapeutic targets. Aromatase, an enzyme that contributes to estrogen synthesis, is localized in cancer tissues, and its inhibitors have been established as typical therapeutic strategies for patients with estrogen-dependent breast cancer [3]. In addition, drugs that block receptors of hormonal action are used to treat breast and prostate cancers. However, several patients show resistance to these hormone therapies, and overcoming this resistance has become a major issue. Although the characteristics of hormone dependence in endometrial and ovarian cancers have been studied, antihormone therapy has not yet been established as the standard treatment.

Controlling estrogen receptor (ER) signaling suppresses the activity of breast cancer cells. ERs contain various structurally similar substances to ligands, in addition to natural estrogen. Tamoxifen, a selective estrogen receptor modulator, is widely used as an anti-estrogenic drug, and fulvestrant, a selective estrogen-receptor-degrading drug, has been approved for the treatment of ER-positive breast cancer. In addition to compounds used as pharmaceuticals, various estrogen-like substances such as endocrine-disrupting chemicals and phytoestrogens have been widely studied. Metals, such as aluminum, cadmium, nickel, and lead, which bind to estrogen receptors and cause estrogenic effects, are called metalloestrogens. Strumylaite et al. [4] reported that urinary cadmium levels were associated with the risk of hormone-receptor-positive and HER2-type breast cancer in an epidemiological study of approximately 500 patients with breast cancer. Kisková et al. [5] reviewed the potential of cannabinoids in breast cancer therapy. In their article, some medical cannabis products were shown to have estrogenic activity, and interestingly, tamoxifen has a high affinity for the G-protein-coupled cannabinoid receptor. A variant form of ERα, ERα36, is known to cause non-genomic signaling of the ER. Nagel et al. [6] demonstrated that high levels of wild-type ERα66 were associated with a favorable prognosis, whereas high ERα36 levels were associated with a poor prognosis in patients with breast cancer. These findings deepen our understanding of the diversity of ER-binding factors and their underlying mechanisms of action.

Okano et al. [7] showed that ER-positive breast cancers with high androgen receptor levels had fewer tumor-infiltrating lymphocytes, lower cytolytic activity, and less responsiveness to neoadjuvant chemotherapy; however, their patients had higher survival rates. Hachim et al. [8] investigated the significance of co-expression of the epithelial–mesenchymal transition suppressor, prolactin receptor (PRLR) and its inducible receptors, and transforming growth factor β receptors (TGFβRI and TGFβRII) in breast cancer. High expression of the PRLR/TGFβRI/TGFβRII gene signature was shown to be indicative of low-grade tumors and a marker of favorable patient prognosis. Studies on crosstalk between ER and androgen receptor (AR) have found an antagonistic effect of the AR on ER transcriptional activity in breast cancer cells [9]. Loss of ERα expression has been reported to trigger epithelial–mesenchymal transition and promote breast cancer cell migration and invasion [10]. Therefore, research on the crosstalk between the ER and receptors for physiologically active substances, including hormones, is required to further elucidate ER signaling in breast cancer cells. Cancer tissue is composed of parenchymal cancer cells and surrounding stromal cells; the intratumoral stroma includes fibroblasts, adipocytes, inflammatory cells, and capillaries [1]. Adipose stem cells (ASCs) from obese donors secrete abundant leptin and increase ERα and aromatase expression in breast cancer [11]. Sabol et al. [12] showed that ASCs from obese donors promoted tumor growth, the metastasis of wild-type ER breast cancer cells, and the metastasis of mutant ER breast cancers. These findings indicated that ASCs derived from obese donors exert both ER-dependent and ER-independent signals in breast cancer cells. The extracellular matrix is an important component of the cancer microenvironment. Fucose is a sugar-chain-constituting monosaccharide, and fucosylated sugar chains are one of the sugar chain modifications most closely related to cancer and inflammation. Herrera et al. [13] implicated core fucosylated N-glycans in the malignant behavior of breast cancer and demonstrated the potential of core fucose modifications of N-glycans as therapeutic targets for patients with breast cancer.

Endometrial carcinoma, the most common type of endometrial cancer, is classified as G1, G2, or G3, according to the morphology of the adenocarcinoma component. Serous carcinomas are the most common uterine non-endometrioid adenocarcinomas. G1 and G2 are classified as type I endometrial cancers; estrogen is thought to be involved in their pathogenesis and development. G3 and serous carcinomas are type II endometrial cancers with a low estrogen association. ER expression in endometrial cancer is known to be relatively high in G1 cases compared with that in other histological types. Similar to breast cancer, the progesterone receptor (PR) is detected in endometrial cancer, and medroxyprogesterone acetate is used for drug therapy. PRs have been detected in endometrial and breast cancers. Although medroxyprogesterone acetate is used for drug therapy, there is no established antiestrogen therapy for patients with endometrial cancer. Therefore, a further multifaceted exploration of estrogen signaling is required to establish anti-estrogen therapies for endometrial cancer.

2-methoxyestradiol, an endogenous metabolite of 17β-estradiol with low affinity for the ER, has been reported to induce apoptosis in various types of cancer cell lines, while being harmless to normal cells [14]. The 2-methoxyestradiol-induced apoptosis pathway is cell-type-specific, although its role in endometrial cancer cells remains unclear. Rincón-Rodriguez et al. [15] investigated 2-methoxyestradiol-induced apoptotic signals in Ishikawa cells, a typical ER-positive endometrial cancer cell line. Both *Spon1* mRNA and F-spondin protein, encoded by *Spon1*, increased only at concentrations of 2-methoxyestradiol that induced apoptosis in Ishikawa cells. The increase in F-spondin/*Spon1* by 2-methoxyestradiol is ER-independent and requires further validation in ER-negative endometrial cancer cell lines. Recently, endometrial cancer has been classified into four molecular prognostic groups using the TCGA database [16]. Therefore, it is necessary to further analyze the hormone dependence of endometrial cancer using a novel grouping system.

Epithelial ovarian cancer is the most common type of ovarian cancer and is mainly classified into serous, clear-cell, endometrioid, and mucinous carcinomas. Serous carcinomas are more common in epithelial ovarian cancers than in other histological types. Ovarian serous carcinomas are classified as high and low grades, mostly high grade. High- and low-grade serous carcinomas exhibit different oncological characteristics, including genetic mutations. O-glycosylation is essential for physiological and pathological functions, such as mucin biosynthesis and proteoglycan core protein integrity. Changes in O-glycan expression in cancer result from alterations in the expression of the N-acetylgalactosaminyltransferase (GalNAc-T) family. Sheta et al. [17] focused on GalNAc-T in epithelial ovarian cancer and investigated the involvement of GalNAc-T3 and 6 (GALNT3 and GALNT6) in malignant behavior both in vitro and in vivo. The GALNT3/T6 double-suppressed ovarian cancer cell line inhibited cell proliferation, migration, and invasion, and increased the survival rate in mice xenografted with the cell line. Nagasawa et al. [18] compared the genetic characteristics of ovarian high-grade serous and clear-cell carcinomas in detail using transcriptome analysis, followed by informatic analysis. Clarifying the characteristics of each histological type of epithelial ovarian cancer at the transcriptional level is expected to lead to the development of tissue type-specific targeted therapies. Furthermore, a similar analysis of commonly used cultured cell line models will lead to the construction of more appropriate in vitro models suitable for different histological types.

ER levels assessed by immunohistochemistry are high in high- and low-grade serous and endometrioid carcinomas but low in mucinous and clear-cell carcinomas [19]. Various clinical studies have suggested the potential of anti-estrogen therapies, such as tamoxifen and aromatase inhibitors, for epithelial ovarian cancer. However, similar to endometrial cancer, there is no standardized hormone therapy for ovarian cancer. Investigating the relationship between the various malignant behavior-related factors described above and the ER may clarify the significance of hormone dependence in ovarian cancer.

Hormone therapy for prostate cancer suppresses androgen production or blocks its action. However, the effects of hormone therapy for prostate cancer are not permanent, and the duration of the first hormone therapy is approximately three years. Prostate cancer that is refractory to hormone therapy is known as castration-resistant prostate cancer. Chemotherapy, the standard for which is docetaxel, is used for castration-resistant prostate cancer. Docetaxel becomes ineffective in many cases, and cabazitaxel has been used in such cases. Sekino et al. [20] reported that in castration-resistant prostate cancer, high TUBB3 expression is involved in both docetaxel and cabazitaxel resistance, and the concomitant use of phosphoinositide 3-kinase inhibitors is effective.

Stoykova and Schlaepfer [21] summarized, in their review, the involvement of proliferating lipid metabolites in the endocrine resistance of prostate cancer. Khurana and Sikka [22] showed that crosstalk between SOX9, AR, and Wnt/β-catenin signaling is involved in the development of castration-resistant prostate cancer, and suggested in their review article that sulforaphane and curcumin, which simultaneously target these signals, have therapeutic benefits. Laufer-Amorim et al. [23] analyzed the genomic profile of AR-negative canine prostate cancer and suggested its potential as a model for human prostate cancer because of its similarities to human prostate cancer.

Research on the role of the AR in hormone-dependent cancers has evolved into a therapeutic target for prostate cancer. In recent years, androgen action in breast and endometrial cancers has become common knowledge [24,25]. To elucidate the androgenic actions, studies have been conducted with prostate as the model initially; however, after these comparative studies, the specific androgenic action of each organ was elucidated. A cross-sectional study of hormone-related cancers will generate new therapeutic targets for each cancer. It is hoped that this study, entitled “Hormone-Dependent Cancers: New Aspects on Biochemistry and Molecular Pathology”, will contribute to the development of new research.
